# Culture-independent metagenomics supports discovery of uncultivable bacteria within the genus *Chlamydia*

**DOI:** 10.1038/s41598-017-10757-5

**Published:** 2017-09-06

**Authors:** Alyce Taylor-Brown, Labolina Spang, Nicole Borel, Adam Polkinghorne

**Affiliations:** 1 Centre for Animal Health Innovation, Faculty of Science, Health, Engineering and Education, University of the Sunshine Coast, Sippy Downs, Queensland Australia; 2 Institute of Veterinary Pathology, University of Zurich, Zurich, CH-8057 Switzerland

## Abstract

Advances in culture-independent methods have meant that we can more readily detect and diagnose emerging infectious disease threats in humans and animals. Metagenomics is fast becoming a popular tool for detection and characterisation of novel bacterial pathogens in their environment, and is particularly useful for obligate intracellular bacteria such as *Chlamydiae* that require labour-intensive culturing. We have used this tool to investigate the microbial metagenomes of *Chlamydia*-positive cloaca and choana samples from snakes. The microbial complexity within these anatomical sites meant that despite previous detection of chlamydial 16S rRNA sequences by single-gene broad-range PCR, only a chlamydial plasmid could be detected in all samples, and a chlamydial chromosome in one sample. Comparative genomic analysis of the latter revealed it represented a novel taxon, *Ca*. Chlamydia corallus, with genetic differences in regards to purine and pyrimidine metabolism. Utilising statistical methods to relate plasmid phylogeny to the phylogeny of chromosomal sequences showed that the samples also contain additional novel strains of *Ca*. C. corallus and two putative novel species in the genus *Chlamydia*. This study highlights the value of metagenomics methods for rapid novel bacterial discovery and the insights it can provide into the biology of uncultivable intracellular bacteria such as *Chlamydiae*.

## Introduction

Recent advances in culture-independent molecular methods and diagnostics, coupled with an increased breadth and depth of sampling, have played a significant role in detecting emerging disease threats in humans and animals^1–4^. This method is advantageous over traditional single-gene pathogen detection methods in which nucleotide differences alone are, in some cases, not powerful enough to distinguish between closely related species^5–7^. Metagenomic sequencing also addresses challenges encountered in a clinical setting: a) if the putative pathogen is novel or unknown, b) if there are no established culture systems, or c) if culture is time-consuming and laborious (eg. for obligate intracellular pathogens such as *Chlamydiae)*.

We recently used metagenomics to sequence and characterise the genome of a putative novel Candidatus species, *Ca*. Chlamydia sanzinia^8^, originating from a diverse group of chlamydial strains circulating among clinically healthy, captive snakes in Switzerland^9^. This species is closely related to *Chlamydia pneumoniae* and its genome encodes several chlamydial virulence markers such as a type three secretion system, translocated actin-recruiting phosphoprotein (Tarp) and chlamydial protease-like activity factor (CPAF)^8^. Elsewhere, chlamydiosis has been described in both wild and captive reptiles, including crocodiles, lizards and snakes, in broad geographical locations, with the impact of infection ranging from asymptomatic infections to severe disease^9–16^. Little or nothing is otherwise known about the the biological diversity of chlamydiae infecting these hosts.

Further, few studies have used metagenomics to describe the metagenome and microbiota of wild or non-model vertebrates, with most studies focussing on mammalian species and agriculturally important animals^17, 18^. Recently, groups have used culture-dependent and independent methods to characterise the microbiota of several anatomical sites in various reptile hosts, to uncover the diversity and function within these communities, and their potential impact on animal and human health^19–23^. We therefore aimed to assess the microbial diversity in *Chlamydia*-positive cloaca and choana samples from captive, asymptomatic snakes. In doing so, we also showed that metagenomics analysis is not only useful for novel chlamydial species discovery but that it also reveals key genomic differences between a novel chlamydial taxon and established species.

## Results and Discussion

### Snake choana and cloaca metagenome assembly and microbial composition

135,167,964 reads were obtained across two cloaca (G1/1679-8 and G2/2464-204) and three choana (G3/2742-324, G6/0661-435, and G7/2741-436) samples in which novel *C*. *pneumoniae* strains were detected by 16S rRNA sequencing. Reads were trimmed for quality and adapter sequence prior to *de novo* assembly and metagenome binning using SPAdes and MaxBin, respectively. 27,763–378,622 contigs were obtained for the samples (76–10,516 contigs over 1,000 bp) (Supplementary Information Table 1).

**Table 1:** Microbial composition of choana and cloaca samples from captive snakes.

	G1/1679-8	G2/2464-204	G3/2742-324	G6/0661-435	G7/2741-436
Host (Family)	*Vipera a*. *ammodytes* (Viperidae)	*Vipera latastei* (Viperidae)	*Corallus batesii* (Boidae)	*Atheris squamiger* (Viperidae)	*Eunectes notaeus* (Boidae)
Anatomical site	Cloaca	Cloaca	Choana	Choana	Choana
No. partial or complete bacterial genomes detected	4	1	3	0	2
No. 16S rRNA sequences detected	5	1	2	0^	2
No. microbial eukaryotes detected	1	0	0	0	1
Most abundant bacterial taxon (coverage of 16S rRNA sequence) (Phylum)	*Serratia marcescens* (~125x) (Proteobacteria)	*Salmonella enterica* ^72x^ (Proteobacteria)	*Chitinophageaceae bacterium* (~702x) (Bacteroidetes)	N.a	*Chitinophageaceae bacterium* (~20x) (Bacteroidetes)

^Only contigs ≥1,000 bp were considered.

N.a; not applicable.

Metagenomic assessment revealed a high level of complexity in the samples, with such deep sequencing allowing us to simultaneously uncover the microflora of these sites, i.e. both putative novel bacteria and microbial eukaryotes residing in the choana and cloaca of these snakes. The cloaca samples harboured up to five bacterial species, and the choana samples up to two species based on recovery of full 16S rRNA sequences and partial or complete bacterial genomes (Table 1).

Many of the BLAST hits of microflora species were known members of reptilian, piscine or mammalian microbiomes, for example *Achromobacter sp*. and *Luteimonas sp*. in the respiratory tract^21, 22^ and *Serratia marcescens*, *Pseudomonas aeruginosa* and *Salmonella enterica* in the cloaca^23^. Interestingly, *Chitinophageaceae* appeared to dominate the choana samples, which has not been described before. This discrepancy is most likely due to the different detection methods used^19–23^. It is unclear what role these bacteria are playing at these sites: *S*. *enterica* has been repeatedly described as a reptile pathogen and such a high level of abundance may provide evidence for this (Table 1). As the name suggests, the *Chitinophagaceae* members in the choana samples (which are 98.5% identical to each other) are rich in chitinases. The role for these bacteria and their enzymes in the choana/oral cavity are unknown, but they may contribute to the digestion of the exoskeleton of animals ingested as part of the snake’s diet. Interestingly, previous studies of reptile microbiota did not detect any *Chlamydia* species^19–23^. This highlights a strength of metagenomics and strongly suggests that it may be a pathogen rather than a commensal, however, additional *in vitro* and *in vivo* studies are obviously necessary to confirm this. We also detected rRNA sequences from a flagellate, *Monocercomonas coluborum*, in a choana sample (G1/1679-8), and a fungal species related to *Sporothrix schenckii* in a cloaca sample (G7**/**2741-436). Further metagenomic sequencing would clarify the presence, abundance and roles of these and other species in the choana and cloaca of snakes.

Despite methylated DNA depletion prior to MDA and sequencing, a host mitochondrial genome was recovered from each sample. It has been shown that mitochondrial DNA may not be methylated in all species^24^, and this is reflected in our data by the presence of these sequences at differing levels of coverage in each sample, combined with fragmented mitochondrial genomes for two species. The mitochondrial genomes were obtained on a single contig or over up to six contigs, with the read coverage ranging from 43x to 38,621x, accounting for ~0.07% to ~31% of the reads (Supplementary Information Table 2).

**Table 2:** Genome characteristics of Uncultured Chlamydia sp. G3/2741-324 compared to closely-related chlamydial species.

	*Ca*. Chlamydia corallus G3/2742-324	*Ca*. C. sanzinia G4/2742-308 (CP014639)	*C*. *pneumoniae* LPCoLN (CP006571.1)	*C*. *pecorum* MC/Marsbar (NZ_CM002310.1)	*C*. *trachomatis* A/HAR-13 (NC_007429.1)
Host	Emerald tree boa (*Corallus batesii*)	Madagascar tree boa (*Sanzinia madagascariensis*)	Koala (*Phascolarctos cinereus*)	Koala (*Phascolarctos cinereus*)	Human *(Homo sapiens)*
Chromosome length (bp)	1,196,452	1,113,233	1,241,024	1,230,439	1,044,459
GC content (%)	39.9	38.5	40.6	40.6	41.2
No. CDS	1,076	998	1,095	1,116	954
No. hypothetical proteins	356	314	426	297	294
Plasmid length (bp)	7,621	7,504	7,530^	7,547^	7,510^
No. CDS on plasmid	8	8	8	8	8

**^**Not present in all strains.

### Chlamydial genome construction from a snake choana metagenome

Given the fact that more than one bacterial genome was present in these metagenomes, a full chlamydial genome could only be recovered from a single sample (G3/2472-324), the characteristics of which are summarised in Table 2 in comparison with other chlamydial genomes. The single metagenome containing chlamydial chromosomal contigs contained 4,445 contigs over 1,000 bp, seven of which were chlamydial and divided between six predicted chromosomal contigs and one predicted plasmid contig. The combined chromosomal contigs total 1,196,452 bp in length and were predicted to encode 1,076 genes. The GC content of 39.33% was comparable to other chlamydial genomes (Table 2). The plasmid contig was 7,522 bp, harboured the typical eight open reading frames and has a lower GC content than the chromosome (32.0%), as is expected for plasmids. The average read coverage across the chromosome and plasmid were ~110x and ~40,134x, respectively.

For the remaining four samples, despite lacking a chlamydial chromosome, a plasmid sequence could be recovered, presumably since chlamydial plasmids are found at copy numbers of two to ten times that of the chromosome^25, 26^ and because MDA may preferentially amplify the plasmid DNA^8^. The plasmid sequences ranged from 7,210 to 7,534 bp, with 5-14,471x average read coverage (Table 3).

**Table 3:** Characteristics of chlamydial plasmids obtained from snake choana and cloaca metagenomes.

	G1/1679-8	G2/2464-204	G3/2742-324	G6/0661-435	G7/2741-436
No. contigs	1	1	1	4	1
Length (bp)	7,534	7,518	7,522	7,210^1^	7,522
Mean read coverage	~3,243x	~389x	~40,132x	~5x	~14,471x
No. ORFs	8	8	8	8	8
BLAST hit (% nucleotide ID)	*C*. *pneumoniae* LPCoLN plasmid (81%)	*C*. *pneumoniae* LPCoLN plasmid (88%)	*C*. *pneumoniae* LPCoLN plasmid (88%)	*C*. *pneumoniae* LPCoLN plasmid (86%)	*C*. *pneumoniae* LPCoLN plasmid (87%)
ArrayTube result	*Chlamydia sp*.	Inconclusive	*C*. *pneumoniae*	*C*. *pneumoniae*	*C*. *pneumoniae*

^1^Possibly incomplete sequence; truncated helicase gene predicted over ends of contig.

^2^Nucleotide identity (%) of near-full length 16S rRNA sequence.

### Phylogenomic analysis of *Ca.* Chlamydia corallus within the *Chlamydiaceae*

**Figure 1:**
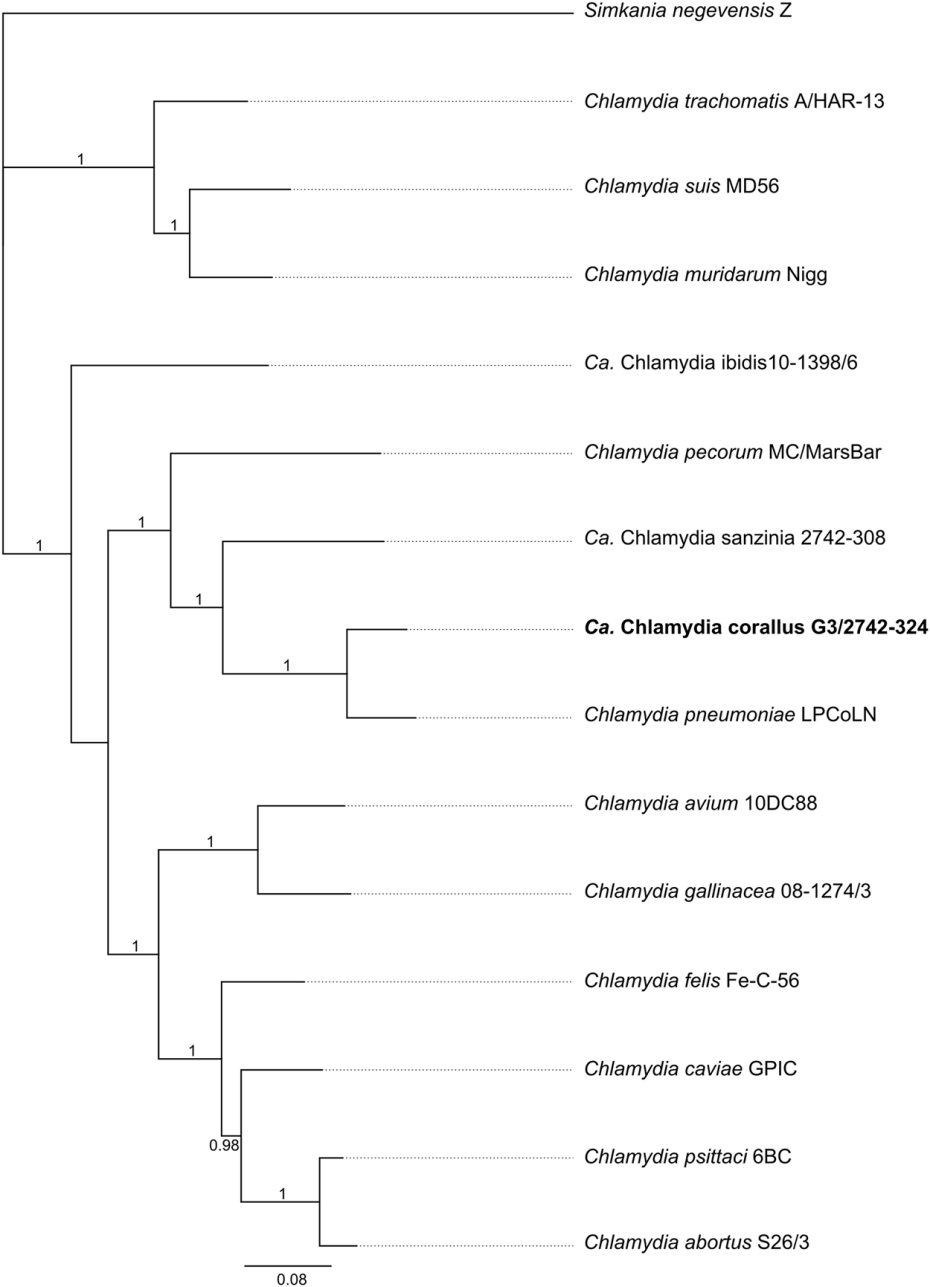
Phylogenetic position of the novel taxon, *Ca*. Chlamydia corallus G3/2742-324 within the *Chlamydiaceae*. Phylogenetically informative marker genes were retrieved from each genome, concatenated and aligned using MAFFT, prior to tree construction using FastTree. Numbers on the branches indicate support values.

To assess the genetic relationship of *Ca.* Chlamydia corallus to other chlamydial species, we utilised the classification scheme published by Pillonel *et al*.^5^. Sequence homology within the 16S rRNA gene placed this novel taxon as a member of the order *Chlamydiales*, (99.2% identical to *C*. *pneumoniae* LPCoLN^9^). Sequence analysis of the additional genes show that this sample is closely related to *C*. *pneumoniae* but is sufficiently genetically different (ANI 90.16-90.31% with *C*. *pneumoniae* AR39 and LPCoLN) that, according to this scheme, G3/2742-324 should be classified as a novel species within the genus *Chlamydia* (Supplementary Information Table 3). To visualise the genetic relationships between this putative new chlamydial species with other members of the genus *Chlamydia*, a phylogenetic tree was constructed from a concatenation of the eleven gene alignments^5^. The resultant phylogenetic tree reiterates the distinct lineage formed by G3/2742-324, in a major cluster with *Ca*. Chlamydia sanzinia, *C*. *pneumoniae* and *C*. *pecorum* (Fig. 1).

Based on the nucleotide identities of the analysed genes highlighted in the classification scheme^5^, G3/2742-324 should be classified as a novel Candidatus species within the genus *Chlamydia*. We propose for it the name *Candidatus* Chlamydia corallus (strain G3/2742-324), so named for the genus of the Amazon Basin emerald tree boa (*Corallus batesii*) in which it was detected. *Ca*. Chlamydia corallus was detected in the choana of a clinically healthy, captive snake in Switzerland.

### Plasmid-based diversity within chlamydial species infecting snakes

Although culture-independent genome sequencing failed to resolve whole genome sequences for the all samples in this study, an extra-chromosomal plasmid was detected in all five metagenomes. An approximately 7.5 Kbp contig from G1/1679-8, G2/2464-204, G3/2742-324, G7/2741-436, and an approximately 7.2 Kbp contig from G6/0661-435, showed a BLASTn hit against the *C*. *pneumoniae* LPCoLN plasmid. The sequence homology between these sequences and *C*. *pneumoniae* LPCoLN plasmid was 78.76–86.09% and among each other was 77.45–96.8%.

Almost all chlamydial species, but not all strains, are known to carry a plasmid, and the nucleotide and amino acid sequences are highly conserved between species^26^. The presence of a plasmid has been suggested to contribute to the pathogenicity or tissue tropism of the chlamydial species^27, 28^, and plasmid proteins are used for diagnostic targets and vaccine candidates. The chlamydial plasmid is normally organised with eight open reading frames (ORFs), encoding for genes involved in plasmid maintenance and glycogen synthesis^29^. Alignment of the plasmid sequences revealed conservation of these ORFs, with the exception of a gap in the coding region for helicase in the sequence for G6/0661-435, which resulted in a partial plasmid sequence (data not shown).

**Figure 2:**
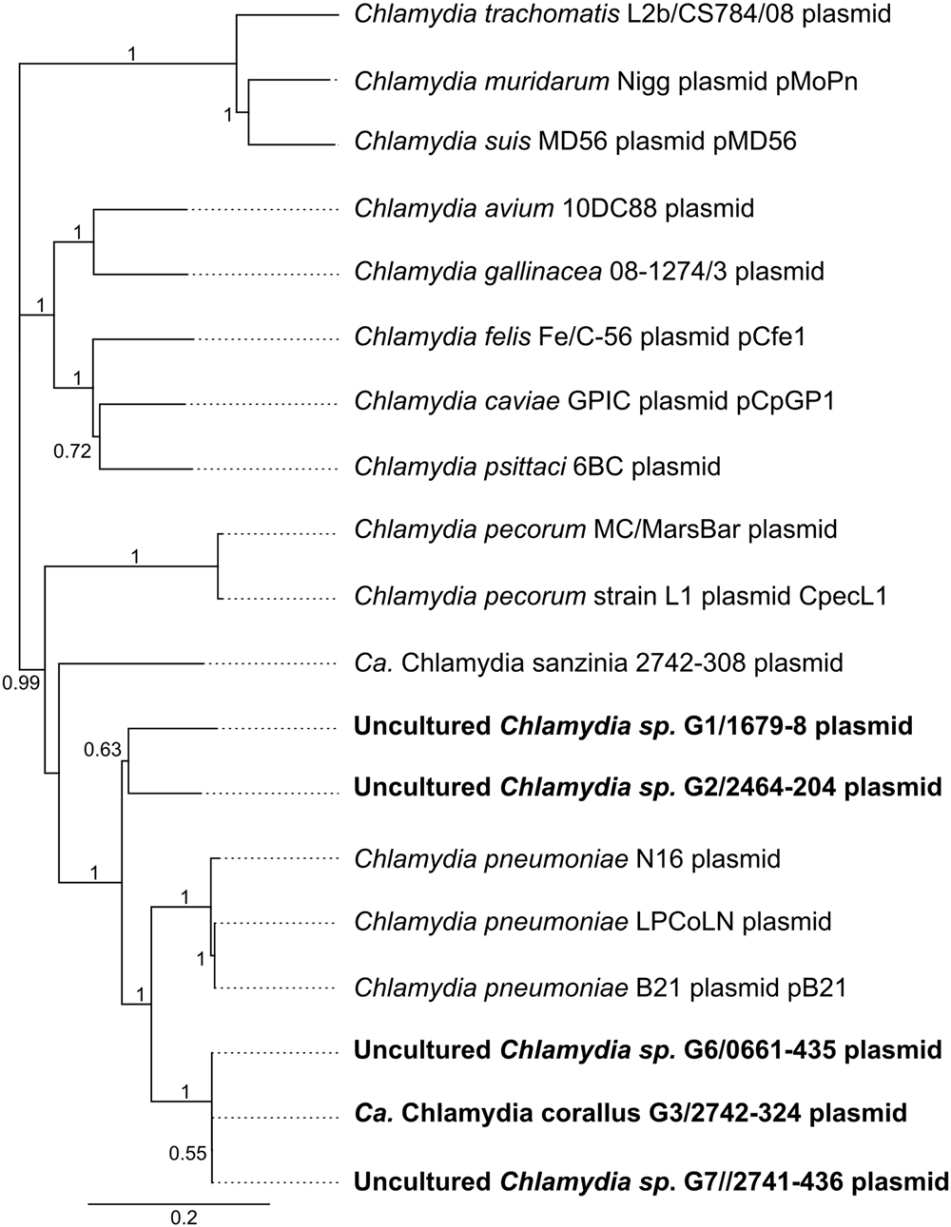
Plasmid sequence-based phylogenetic analysis of all samples sequenced in this study and other chlamydial plasmid sequences. Nucleotide sequences were aligned using MAFFT and tree was constructed using FastTree. Support values are shown on the branches.

Previous research has also shown that there is a co-evolution between the chromosome and plasmid sequences for the chlamydial species^26, 30^, so in the absence of chlamydial chromosomal genetic data for these additional samples, we performed phylogenetic analysis on the nucleotide sequences across the plasmid in order to assess the genetic relationship of all strains obtained from the metagenomes in this study. In agreement with the tree constructed from its chromosomal loci, phylogenetic analysis revealed that *Ca*. Chlamydia corallus clusters with but is genetically distinct from *C*. *pneumoniae*. (Fig. 2). Plasmid sequences from G6/0661-435 and G7/2741-436 can also be found in this clade. Notably, G1**/**1679-8 and G2/2464-204 form two additional branches distinct from *Ca*. C. corallus, *C*. *pneumoniae* and *Ca*. C. sanzinia, sharing 84.09% of their nucleotides with each other and 72.27% to 83.51% with these three species.

As no typing scheme exists to distinguish species based on plasmid sequence analysis or phylogeny, we used linear regression analysis to assess the relationships between chromosome and plasmid pair-wise patristic distances (sum of branch lengths) within the *Chlamydiaceae* (Supplementary Information Table 4 and Supplementary Information Fig. 1). Based on the phylogenetic markers used in this study, at the strain level (eg. *C*. *pneumoniae* LPCoLN and N16; *C*. *pecorum* MC/MarsBar and L1), patristic distances for the chromosome are 0, while for the plasmid they are 0.01–0.02. For closely related species pairs such as *C*. *caviae* and *C*. *felis* or *C*. *suis* and *C*. *trachomatis*, chromosomal and plasmid patristic distances are 0.12–0.16 and 0.20–0.22, respectively. For more distantly related species pairs such as *C*. *trachomatis* and *C*. *psittaci*, or *C*. *pecorum* and *C*. *muridarum*, chromosomal and plasmid patristic distances are higher: 0.28–0.33 and 0.52–0.57 (Supplementary Information Table 4). For the sequences obtained in this study, branch lengths between G6/0661–435 and G7/2741–436, and between these two samples and G3/2742–324, are equivalent to those at the strain level (0.0), as are their extrapolated chromosomal patristic distances based on the curve (Supplementary Information Table 4, Supplementary Information Fig. 1). On the other hand, the plasmid and extrapolated chromosomal patristic distances between G1/1679-8, G2/2464-204 and *C*. *pneumoniae* of 0.18–0.21 and 0.11–0.13, respectively, are slightly lower than those of *C*. *caviae* and *C*. *felis*, but not as close as that of *C*. *psittaci* and *C*. *abortus*, highlighting their relatedness to each other (Fig. 2). Meanwhile, their branch lengths with most other members of the genus is 0.36–0.41, which is comparable to most other pair-wise distances (Supplementary Information Table 4), thus may represent two distinct novel species.

These data also fit with initial testing results, in which G1/1679-8 and G2/2464-204 could not be definitively assigned to a species based on a *Chlamydiaceae* ArrayTube assay designed to detect established species^8^. G1/1679-8 was identified as a *Chlamydia* species, and G2/2464-204 did not yield a conclusive result (Table 3)^9^. G3/2742-324, G6/0661-435 and G7/2741-436 were designated as *C*. *pneumoniae*, which is in line with their close plasmid nucleotide identity. This suggests the assay is less specific when taxa are so closely related, but is robust enough to detect novel species.

Given the above, the phylogenetic position of G1/1679-8 and G2/2464-204 among other species and their evolutionary distances from other species and strains provide strong evidence of additional species-level diversity within the *Chlamydiaceae*. These data provide (a) evidence that, for some taxa, 16S rRNA sequencing is not sufficient to speciate, (b) validation of the use of genome sequencing to further investigate genetic diversity within and/or between populations, and (c) evidence for the use of plasmid sequence to assess diversity and phylogeny of novel chlamydial species for which plasmids are ubiquitous.

### Genetic differences within the plasticity zone of Ca. *Chlamydia corallus*

In order to further characterise the genome of the novel species, *Ca*. Chlamydia corallus, in comparison to other chlamydial species, the plasticity zone (PZ) region was analysed. The plasticity zone is a unique region within the *Chlamydia* genome that has been associated with host adaptation for some chlamydial species^6, 31^. The well-known variability between the chlamydial species within this region makes it an appropriate target for understanding the factors that might have influenced the tissue tropism of *Ca*. Chlamydia corallus.

**Figure 3:**
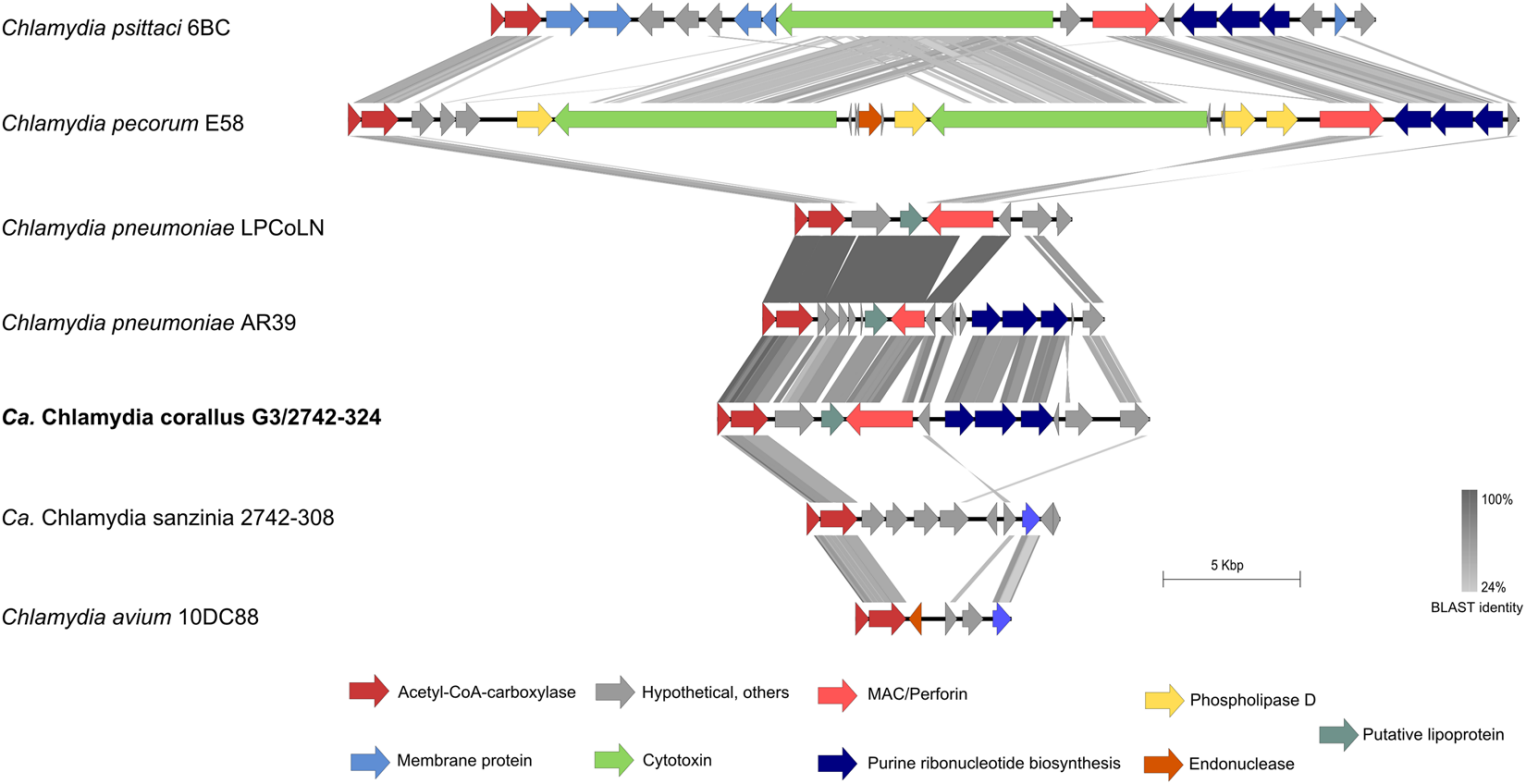
Comparison of plasticity zone regions encoded by *Ca*. Chlamydia corallus and related chlamydial species. Figure constructed using Easy Fig^54^. Grey shading represents tBLASTx matches (see BLAST identity scale). Coloured arrows represent coding regions (see legend).

The plasticity zone of *Ca*. Chlamydia corallus is approximately 13,700 bp in size and composed of genes required for several biochemical pathways such as Acetyl-CoA-carboxylase (*accBC)*, purine and pyrimidine synthesis genes (*guaABadd*) and the MAC/perforin gene, as seen in Fig. 3. When compared with other chlamydial species, the plasticity zone harboured by *Ca*. Chlamydia corallus is structurally most similar to the human-isolated strain of *C*. *pneumoniae* AR39 (Fig. 3). Both species have a slightly smaller plasticity zone than other species, due to the absence of any cytotoxin, which is present in *C*. *psittaci* and duplicated in *C*. *pecorum* ^32, 33^. The main difference between the PZs of *Ca*. C. corallus and *C*. *pneumoniae* appears to be fragmented or truncated hypothetical proteins in AR39, and the absence of a putative lipoprotein in *Ca*. Chlamydia corallus, which is present in both strains of *C*. *pneumoniae* (Fig. 3)^34^.

Compared to the other known chlamydial species infecting snakes, the plasticity zone of *Ca*. Chlamydia corallus was genetically variable from *Ca*. Chlamydia sanzinia (Fig. 3). For instance, the MAC/perforin complex gene was not detected in the plasticity zone of *Ca*. Chlamydia sanzinia^8^. The function of the MAC/perforin gene in the chlamydial species is unknown, but has been suggested to contribute to the pathogenesis of these species^35^. Additional differences in the PZs of *Ca*. Chlamydia sanzinia and *Ca*. Chlamydia corallus lie in the purine ribonucleotide biosynthesis pathways, as highlighted in Fig. 3. The purine ribonucleotide biosynthesis (*guaABadd*) cluster, detected in *Ca*. Chlamydia corallus, plays a critical role in both *de novo* and salvage pathways for purine synthesis in prokaryotes^36^. This cluster is present in some chlamydial species^33, 34, 37^, but was never detected in the other recently described snake chlamydia, *Ca*. Chlamydia sanzinia^8^. Chlamydial species that do not encode for this gene cluster are most likely able to synthesise purines through alternative pathways^38^. Its absence in other bacterial species, such as *Helicobacter pylori*, has been found to have an effect on their rate of growth^36^. The absence of the *guaABadd* genes in several of the chlamydial species, however, indicates that these genes are not needed by the chlamydial species; its absence in *Ca*. Chlamydia sanzinia also suggests that these genes are not necessary for the chlamydial species to establish infection in snakes^8, 37^.

No tryptophan operon (*trpAB)* was detected in the plasticity zone or other genomic regions of *Ca*. C. corallus. Tryptophan is a necessary amino acid for chlamydial growth^39^, however, host cell defence mechanisms against chlamydial infections exist in which interferon gamma (IFN-γ) production depletes intracellular tryptophan stores^40^. Certain strains of *C*. *trachomatis* encode for an intact tryptophan operon (*trpAB*), which is absent or incomplete in other chlamydial species^37^, suggesting that not all chlamydial species are able to synthesise tryptophan. For urogenital strains of *C*. *trachomatis*, the vaginal microflora is believed to provide indole, allowing for synthesis of tryptophan^39^. The absence of a tryptophan operon would suggest that *Ca*. C. corallus either has alternative pathways for synthesising tryptophan or is possibly completely auxotrophic for tryptophan. Notably, neither *Ca*. C. corallus nor Ca. C. sanzinia encode for an aromatic amino acid hydroxylase, which has been suggested to contribute to tryptophan metabolism in the absence of *trpAB*. As has been suggested for *C*. *trachomatis* ^39^, the diverse microflora in these snakes may provide nutrients or substrates for chlamydial synthesis of amino acids. Metagenomic mining revealed several tryptophan synthesis pathway or rescue genes encoded by the bacteria present in the cloaca and choana samples, for example, tryptophan synthase, tryptophanase and indole-3-glycerol phosphatase were detected among the samples, encoded by *Achromobacter sp*., *Serratia marcescens*. *Clostridium sp*.*, Salmonella enterica* and *Chitinophagaceae*. Previous studies also describe the presence of indole-producing bacteria at these sites^21–23^.

In the current study, we have used culture-independent metagenome analysis to investigate the microbial metagenome of snake choana and cloaca samples. In doing so, we have shown that this method provides a wealth of biological information for novel species discovery through microbial community profiling, and have described the presence of highly abundant bacterial species at these sites, some of which have not previously been described. The animal and public health implications of these findings are unknown, but the repeated observations of human pathogens in the microflora of snakes^21, 22^ and other reptiles warrants further investigation.

The metagenomic method used is particularly useful for characterising novel species or strains with no reference genome such as novel uncultivable bacteria (eg. members of the phylum *Chlamydiae*). The complexity within these anatomical sites meant that despite previous detection of chlamydial 16S rRNA sequences by PCR, only a chlamydial plasmid could be detected in all samples, and a single chlamydial chromosome. Nonetheless, comparative analysis of the novel chlamydial species with other *Chlamydia sp*. revealed genetic differences in regards to purine and pyrimidine metabolism. The detection of chlamydial plasmids in all samples, which was only possible using this method, highlights additional diversity within the *Chlamydiae*, which appears to be a growing trend with increased breadth and depth of sampling and advances in molecular techniques. Further studies, such as metatranscriptomic analysis would better elucidate the complex role of the microbiota on chlamydial pathogenesis and vice versa.

## Materials and Methods

### Sample preparation

Suspected novel genotypes (n = 5) of *C*. *pneumoniae* were recently detected in collections of captive snakes in Switzerland^8^. Clinical swabs were taken from either the cloana or choana of clinically healthy snakes, and DNA was extracted as previously described^8^. All samples were subjected to host methylated DNA depletion prior to multiple displacement amplification, as previously described^9^.

### Ethics approval and consent to participate

The collection and molecular analysis of the snake samples was approved and performed in accordance with the relevant guidelines and regulations of the Veterinary Office of Canton Zurich (authorization no. ZH010/15).

### Metagenome assembly and analysis

Deep sequencing was carried out on an Illumina NextSeq at the Australian Genome Research Facility using 150 bp paired-end reads. Read quality was assessed through FastQC v.011.2 and reads were trimmed for adaptors and quality using Trimmomatic v.305^41^. Reads were assembled into contigs using SPAdes v.3.1.1 in metagenome mode with default kmer values (21, 33, 55)^42^﻿. Each assembly was assessed through QUAST^43^. To obtain chlamydial contigs from the assembled metagenome, contigs were subject to BLAST analysis against an in-house chlamydial genome database, and subsequent analysis against the NCBI nucleotide database. Contigs with hits against chlamydial sequences were automatically annotated using RAST^44^ and manually curated in Artemis^45^.

Metaxa was employed initially to assess the species richness within the resulting metagenomes, detecting ribosomal RNA subunits of various origins^46^. MaxBin was used to construct partial or complete draft genomes for the microbial species detected in the samples and determine genome completeness for each assembly^47^.

Burrows-Wheeler aligner, SAMtools and BEDtools were used to map reads and assess read coverage across the various metagenomic components^48–50^.

### Phylogenetic analysis

The genetic relationships of the novel species described in this study to other chlamydial species was assessed using the classification system published by Pillonel *et al*.^5^. Individual genes were extracted from the assembled genome and established chlamydial species, including *Simkania negevensis* as an out group. Extracted genes were concatenated and aligned using MAFFT^51^ and a phylogenetic tree based on the resulting alignment was constructed using FastTree^52^; both were performed in Geneious v7.1^53^.

Plasmid phylogeny was performed based on the alignment of nucleotide sequences using MAFFT^51^ and tree construction using FastTree^52^. In order to include plasmid sequences from all possible species, nucleotide sequences were re-ordered and large gaps were removed so that each resulting plasmid sequence was 5,522–6,170 bp, thus comparable to *C*. *suis* plasmid which lacks the parA and pgp-6 genes.

### Data availability

The metagenomic sequence data obtained for the Ca. Chlamydia corallus chromosome and plasmid was deposited in Genbank under accession numbers as part of bioproject PRJNA312988.

## Electronic supplementary material

Supplementary information
