# Supplementary material for: Culture-independent metagenomics supports discovery of uncultivable bacteria within the genus Chlamydia
Source: Sci Rep. 2017 Sep 6;7:10661. doi: 10.1038/s41598-017-10757-5 (PMC5587560; doi:10.1038/s41598-017-10757-5)
Supplement: Supplementary file 1 — Supplementary information [file 41598_2017_10757_MOESM1_ESM.pdf]

Culture-independent metagenomics supports discovery of uncultivable bacteria within the genus *Chlamydia*

Alyce Taylor-Brown[1], Labolina Spang[1], Nicole Borel[2], Adam Polkinghorne[1#]

Supplementary Information

Table S1: Assembly metrics for snake choana and cloaca metagenomes.

Table S2: Mitochondrial genome metrics.

Table S3: Nucleotide identities between phylogenetic markers of *Ca.* Chlamydia corallus G3-2742-324 and other chlamydial species. Cut-off values in brackets indicate the lower limit to be classified as a different family, genus or species.

Table S4: Chromosome and plasmid pair-wise patristic distances within the *Chlamydiaceae*.

Figure S1: Relationship between chromosome and plasmid pair-wise patristic distances within the *Chlamydiaceae.* Plasmid patristic distances for species pairs were computed and plotted against those of the corresponding chromosomal distances. Pair-wise patristic distances for novel plasmid sequences obtained in this study for which there was no chromosome were then extrapolated from the curve.

Supplemental material; Table S1: Assembly metrics for snake choana and cloaca metagenomes

**Assembly metric**	**G1/1679-8**	**G2/2464-204**	**G3/2742-324**	**G6/0661-435**	**G7/2741-436**
No. contigs (≥ 0 bp)	28,051	44,631	122,400	27,763	378,622
No. contigs (≥ 1000 bp)	3,940	2,606	4,445	76	10,516
Largest contig (bp)	637,828	67,222	547,657	17,580	84,931
GC (%)	55.17	47.31	38.49	41.96	37.73
N50	49,197	3,850	1,538	840	921
L50	184	536	2,056	123	12,445

Supplemental material; Table S2: Mitochondrial genome metrics

**Assembly metric**	**G1/1679-8**	**G2/2464-204**	**G3/2742-324**	**G6/0661-435**	**G7/2741-436**
Host species	*Vipera a. ammodytes*	*Vipera latastei*	*Corallus batesii*	*Atheris squamiger*	*Eunectes notaeus*
No. contigs	1	1	1	1	7
Length of mitochondrial genome (bp)	16,355	16,419	16,599	17,552	16,472
Coverage of mitochondrial genome	~87x	~64x	~38,621x	~43x	~33,695x
% of reads mapped to mitochondrial genome	0.07%	0.03%	31%	0.02%	22%

Supplemental material; Table S3: Nucleotide identities between phylogenetic markers of *Ca*. Chlamydia corallus G3/2742-324 and other chlamydial species.

	**Gene (cut-off value[1])**	***C. pneumoniae* LPCoLN**	***C. pneumoniae* AR39**	***Ca.* C. sanzinia 2742-308**	***C. pecorum* MC/MarsBar**	***S. negevensis* Z**
Family	16S rRNA gene (92.5%)	99.21%	98.83%	97.03%	95.71%	84.46%
	23S rRNA gene (91%)	99.13%	98.92%	97.70%	96.54%	83.81%[2]
Genus	Chromosomal replication initiator; *dnaA* (70%)	90.32%	90.32%	80.71%	78.69%	59.44%
	2-oxoglutarate-dehydrogenase E1 component; *SucA* (64%)	87.28%	87.39%	74.14%	69.71%	49.53%
	Hypothetical protein; *Hyp325* (57%)	81.18%	81.18%	73.15%	73.61%	Np
	Enoyl-[acyl-carrier-protein] reductase; *FabI* (78%)	91.33%	91.33%	76.00%[3]	74.44%[3]	62.06%
Species	RNA polymerase sigma-54 factor; *RpoN* (96%)	85.17%	82.01%	66.05%	59.94%	42.37%
	Cell division protein; *FtsK* (98%)	87.33%	87.33%	71.24%	66.37%	48.73%
	Oligoendopeptidase F; *PepF* (96%)	85.29%	85.35%	70.31%	67.75%	48.52%
	Adenylate kinase; *Adk* (95%)	86.36%	85.36%	65.42%	59.66%	48.89%
	Glutamate-1-semialdehyde 2,1-aminomutase; *HemL* (95%)	83.60%	83.52%	63.92%	59.56%	Np
	Average nucleotide identity	90.31%	90.16%	78.56%	75.71%	56.01%

[1] Values in brackets indicate the upper limit for nucleotide identity between a putative novel taxon and established species for the novel taxon to be classified in the same family, genus or species [14].

[2] After gap removal

[3] Below cut-off value

N.p; Not present

Supplemental material; Table S4: Chromosome and plasmid pair-wise patristic distances within the *Chlamydiaceae*

	**Species pairs**	**Chromosome**	**Plasmid**
	C. pneumoniae LPCoLN, B21, N16, AR39, TW-183	0.00	0.02
	C. pneumoniae LPCoLN, B21, N16 (animal strains)	0.00	0.02
	C. pecorum MC/MarsBar, L1, IPA	0.00	0.01
Reference pairs	*C. pneumoniae* LPCoLN + *Ca*. C. sanzinia 2742-308	**0.23**	**0.33**
	C. pneumoniae LPCoLN + *C. pecorum* MC/MarsBar	0.27	0.39
	C. pneumoniae LPCoLN + *C. trachomatis* A-HAR/13	0.32	0.57
	C. pneumoniae LPCoLN + *C. psttaci* 6BC	0.27	0.41
	C. psttaci 6BC + *C. trachomatis* A/HAR-13	0.29	0.54
	C. caviae GPIC+ *C. muridarum* Nigg	0.28	0.52
	C. pecorum MC/MarsBar + *C. suis* MD56	0.33	0.57
	C. psittaci 6BC + *C. abortus* S26/3	**0.05**	†
	C. avium 10DC88 + *C. gallinacea* 08-1274/3	**0.14**	**0.2**
	C. caviae GPIC + *C. felis* Fe-C-56	**0.12**	**0.21**
	C. trachomatis A/HAR-13 + *C. suis* MD56	**0.16**	**0.22**
	C. trachomatis A/HAR-13 + *C. muridarum* Nigg	**0.15**	**0.21**
This study	G3/2742-324 + *C. pneumoniae* LPCoLN	0.10	0.14
	G3/2742-324 + *Ca.* C. sanzinia 2742-308	0.22	0.33
	G3/2742-324 + *C. pecorum* MC/MarsBar	0.27	0.38
	G3/2742-324 + G7/2741-436	*0.00*	0
	G3/2742-324 + G6/0661-435	*0.00*	0
	G6/0661-435 + G7/2741-436	*0.00*	0
	G3/2742-324 + G1/1679-8	*0.12*	0.2

G3/2742-324 + G2/2464-204	*0.12*	0.19
G1/1679-8 + G2/2464-204	*0.11*	0.18
G1/1679-8 + *C. pneumoniae* LPCoLN	*0.13*	0.21
G2/2464-204 + *C. pneumoniae* LPCoLN	*0.12*	0.19

Bold font; species pairs with which the relationship between the sequences in this study and reference pairs are comparable.

Italics; chromosome patristic distances that were extrapolated from the linear regression curve (y = 1.6776x - 0.0053; R^2 = 0.9605).

† *C. abortus* does not harbor a plasmid, hence no plasmid patristic distance could be determined, nor relationship between chromosome and plasmid patristic distance.

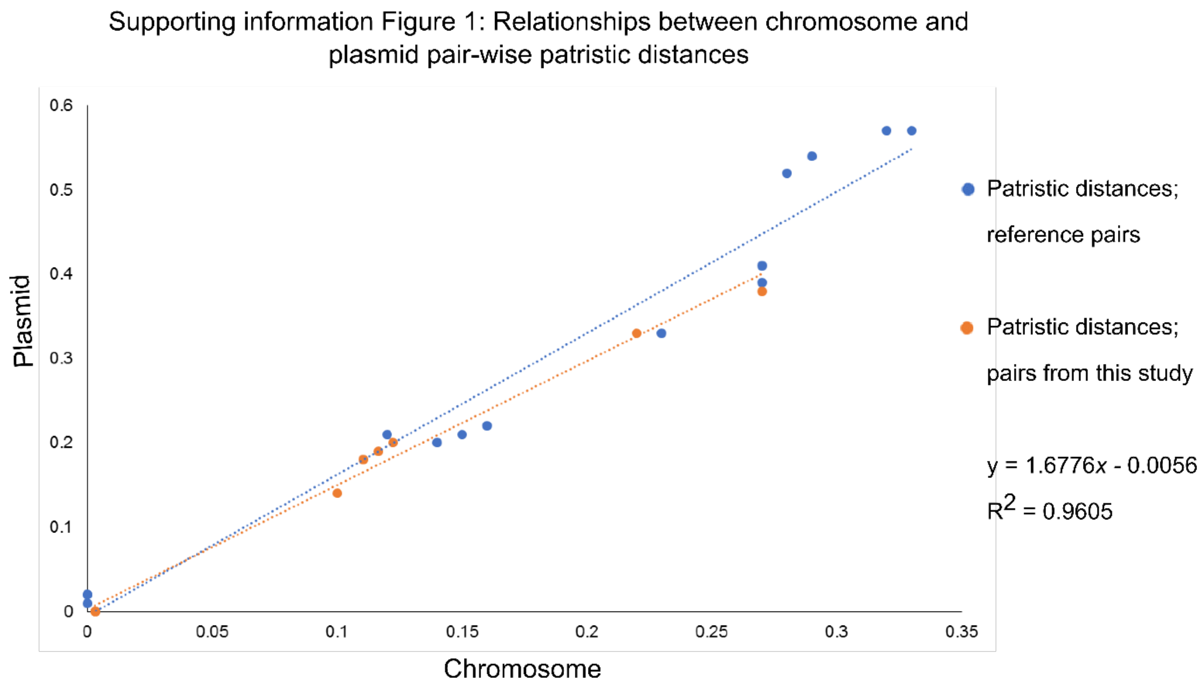

Supplemental material; Figure S1: Relationship between chromosome and plasmid pair-wise patristic distances within the *Chlamydiaceae.* Plasmid patristic distances for species pairs were computed and plotted against those of the corresponding chromosomal distances. Pair-wise patristic distances for novel plasmid sequences obtained in this study for which there was no chromosome were then extrapolated from the curve.
